# Representation of chemistry transport models simulations using knowledge graphs

**DOI:** 10.1186/s13321-025-01025-0

**Published:** 2025-05-31

**Authors:** Eduardo Illueca Fernández, Antonio Jesús Jara Valera, Jesualdo Tomás Fernández Breis

**Affiliations:** 1 Department of Clinical Science, Intervention and Technology, Karolinska Institutet, Stockholm, Sweden; 2 Department of Informatics and Systems, University of Murcia, IMIB-Pascual Parrilla, Murcia, Spain; 3 Libelium Murcia (HOP Ubiquitous S.L), Ceutí, Spain

**Keywords:** Chemical transport models, Air quality simulation, Knowledge graphs, Ontology, CHIMERE-WRF

## Abstract

**Abstract:**

Persistent air quality pollution poses a serious threat to human health, and is one of the action points that policy makers should monitor according to the Directive 2008/50/EC. While deploying a massive network of hyperlocal sensors could provide extensive monitoring, this approach cannot generate geospatial continuous data and present several challenges in terms of logistics. Thus, developing accurate and trustable expert systems based on chemistry transport models is a key strategy for environmental protection. However, chemistry transport models present an important lack of standardization, and the formats are not interoperable between different systems, which limits the use for different stakeholders. In this context, semantic technologies provide methods and standards for scientific data and make information readable for expert systems. Therefore, this paper proposes a novel methodology for an ontology driven transformation for CHIMERE simulations, a chemistry transport model, allowing to generate knowledge graphs representing air quality information. It enables the transformation of netCDF files into RDF triples for short term air quality forecasting. Concretely, we utilize the Semantic Web Integration Tool (SWIT) framework for mapping individuals using an ontology as a template. Then, a new ontology for CHIMERE has been defined in this work, reusing concepts for other standards in the state of the art. Our approach demonstrates that RDF files can be created from netCDF in a linear computational time, allowing the scalability for expert systems. In addition, the ontology complains with the OQuaRE quality metrics and can be extended in future extensions to be applied to other chemistry transport models.

**Scientific contributions:**

Development of the first ontology for a chemistry transport model. FAIRification of physical models thanks to the generation of knowledge graphs from netCDF files. The ontology proposed is published in PURL (https://purl.org/chimere-ontology) and the knowledge graph generated for a 72-h simulation can be accessed in the following repository: https://doi.org/10.5281/zenodo.13981544.

## Introduction

Air quality and atmospheric pollution produces millions of deaths each year and is one of the major health issues according to the World Health Organization (WHO) since 91% of people live in cities where the WHO threshold standards are exceeded [1]. Particularly, airborne pollution can cause several mortal diseases [2, 3]. To improve the air that citizens breathe, Directive 2008/50/EC of the European Parliament and of the Council of 21 May 2008 on ambient air quality and cleaner air for Europe indicates that each member state must classify its territory into air quality zones that constitute an independent and homogeneous geographical unit for air quality management [4]. For this reason, one of the key insights of digital transformation is air quality monitoring, evaluation, prediction and mitigation to create plans for cleaner air. This problem is more serious in cities, which are highly populated as well as very polluted, increasing the exposure of citizens to unhealthy pollutants. Thus, air quality management and monitoring is among the most important services that new expert systems should cover [5].

To tackle this problem, a standardized approach in the literature for pollution assessment is to simulate its dispersion with computational fluid dynamics (CFD), a strategy that models the processes affecting gasses and particles in the atmosphere, which requires a huge amount of computing resources [6]. Inside CFD, chemistry transport models based on a Eulerian approach are the most important, used to perform accurate calculations of the physical and chemical processes in the atmosphere, thereby providing distributions of many different air pollution components on the spatial and temporal resolution needed to describe the variations in exposure, experienced by the individuals of the cohort [7]. One of the most important at mesoscale level is the CHIMERE model, coupled with the meteorological driver WRF [8].

The use of these models generates a wide amount of heterogeneous data that should be integrated in expert systems. In this context, the semantic web provides machine-readable information whose infrastructure is based on the World Wide Web Consortium (W3 C) standards [9], which are also reinforced by the FAIR principles for data management and stewardship, establishing that ontologies should be used for restricting the meaning of knowledge graphs, which are organized representations of real world entities and relationships that provides a context/vocabulary to enrich the data [10]. However, the data provided by chemistry transport models are in standard traditional formats, delaying the use of interoperable data in air quality systems [11]. CHIMERE works with the netCDF data format, a scientific standard for representing multidimensional arrays commonly used with low-level programming languages such as C, C + +, or FORTRAN [12]. This format has limitations in data querying and metadata annotation, which makes it difficult to meet FAIR guidelines. In consequence, further research is needed to ensure the interoperability of netCDF data.

For this reason, this paper aims to generate knowledge graphs representing the results of a CHIMERE simulation keeping the relationships between pollutants and entities, as knowledge graphs have demonstrated to offer a powerful solution for integrating and analyzing complex, fragmented data in cheminformatics, particularly in drug development and chemical safety assessment [13]. We hypothesize that it is possible to transform netCDF files into RDF/XML triples with semantic information by using an ontology-driven approach. This approach requires the definition of an ontology of the chemistry transport models*.* By utilizing knowledge graphs, it becomes possible to reason over pollution datasets, enabling the inference of new knowledge and connecting these datasets with other ontologies and graphs. For example, air quality pollutants, as chemical entities, can be linked to their harmful health effects through toxicological mechanisms or their involvement in metabolic pathways. Our approach extends the utility of air quality data beyond traditional applications, fostering interdisciplinary insights and supporting more informed decision-making in public health and environmental policy in compliance with Directive 2008/50/EC, which defines the threshold values that policy makers should guarantee. In addition, European legislation claims for interconnected air quality networks from different countries, and this paper allows researchers going a step beyond in reusing and sharing air quality data.

Thus, the scientific contribution of this paper relies on the following points: (i) to the best of our knowledge, this is the first approach of transforming netCDF data into RDF knowledge graphs. This format is not only used by CHIMERE but also by many physical systems, so our contribution may also benefit other users of the netCDF format; (ii) we propose the first ontology for the chemistry transport model domain and (iii) we demonstrate that interoperable air quality data can be generated with support of state-of-the-art tools such as the Semantic Web Integration Tool (SWIT) [14], which can also validate that the data is compliant with the constraints of the ontology. In addition, we aim to understand if the knowledge graph generation process is scalable for wider domains and long-term simulations. We have deployed these methods in a use case focused on the Region of Murcia, an area with an important ecological value, agricultural and livestock activities, and medium-scale industry, being representative of the Mediterranean countries [15].

The rest of the paper is structured as follows. First, the *Background* section sets the theoretical background and the work performed in ontology driven transformation and its applications to air quality. Then, the *Materials and Methods* section describes the methodology used for performing the simulation and transforming the netCDF files into knowledge graphs, as well as the architecture and ontology defined. Next, *Results* regarding computation times of the mapping process, evaluation of the ontology and validation of the use case are provided. Next, these results are analyzed in the *Discussion* section and compared with state of the art, obtaining insights for future researchers. Finally, the main conclusions and future challenges are presented in the *Conclusions*.

## Background

Effective and trustworthy data-driven science and sustainability require the use of data at scale and a transition from the current closed and silo-based approaches towards more networked scholarship [16]. In this context, a group of internationally recognized leaders in data management co-authored the FAIR Principles—a set of guiding principles to ensure that contemporary data resources and scholarly output are Findable, Accessible, Interoperable, and Reusable [17]. FAIR compliance is essential to enable powerful new prediction analytics to access data for forecasting computation, understand the semantic context providing provenance of the information, and understand the rules for data transformation [10]. Chemistry Transport Models (CMT) are one of the key elements in the new air quality platforms, and expert systems should work on the FAIRification of these mathematical models [18].

Data transformation methods have been proposed in projects for semantic data exploitation and with XML datasets and relational databases [19]. Regarding XML datasets, some studies describe strategies for transforming XML instances into RDF triplets but do not transform XML attributes [20]. Other approaches convert XML instances into RDF according to a mapping between XSD and OWL. These, called XSD2OWL mappings, are canonical since all the XML files are transformed into RDF by applying the same rules [21, 22]. On the other hand, [23] proposed the transformation of XML into RDF by applying XPath-based mappings. On the relational database side, the W3C RDB2RDF specification proposes a canonical transformation/mapping for relational databases to RDF [24]. In addition, Bio2RDF has become the most prominent initiative for generating biomedical RDF datasets. Bio2RDF has developed RDF versions for 35 datasets (Release 3 July 2014), and its website contains non-canonical transformation scripts for such resources.

From a process perspective, the semantic transformation of data requires the execution of Extraction-Transformation-Load (ETL) processes. Canonical transformation approaches apply the same ETL process to all the data. The required information about the semantics of the data sources is sometimes missing in the data schema or coded in natural language [25], making such canonical processes ineffective enough to obtain semantically rich datasets. For this reason, ontology-driven processes use ontologies to give precise meaning to the source data, which will be made explicit in the transformation phase, enabling consistency checking in the transformation and load phases, which prevents the creation of logically inconsistent content. RDB2OWL [26] and Karma [27] exploit mappings between relational schemas and ontologies to generate knowledge graphs. However, the real meaning of the entities represented is not used in such processes, so new tools based on an ontology-driven approach, compatible with XML and relational databases has emerged, such as Morph-KGC [28], maplib [29], SWIT [14], which verifies the consistency of the data with respect to the ontology constraints, and mapping formats as RML [30].

Regarding the applications of these methodologies, the use of ontologies and knowledge graphs is widely extended in experimental sciences to transform and learn from relational databases [31]. Ontologies are used in different areas, such as knowledge engineering, artificial intelligence, computer sciences, bioinformatics, cheminformatics and environmental impact assessment [32]. In computational and evolutive biology, the major effort in the state of the art is *Gene Ontology* [33]. At the same time, in cheminformatics, the most comprehensive ontology is ChEBI, describing small compounds, most of them environmental and atmospheric pollutants [34]. In the field of chemical processes, the OntoKin ontology for capturing both data and the semantics of chemical kinetic reaction mechanisms has been developed. Such mechanisms can be applied to simulate and understand the behavior of chemical processes, for example, the concentration of pollutants from internal combustion engines [35]. Delving into environmental sciences, the ENVO ontology includes some 2159 classes primarily representing biomes, geographic features, and environmental materials, along with 18,791 axioms (logical statements) defining, interconnecting, and interrelating them [36]. These ontologies have been used to generate RDF datasets by SWIT, which has been tested on colorectal cancer data, orthology data, and chemical compounds data [37].

However, these solutions found in the state of the art have been tested for relational databases and CSV files. The challenge of chemistry transport models is that the output is in netCDF format, which does not fit the relational schema and presents challenges in FAIRification [12]. For this reason, our work goes beyond the state of the art because: i) we propose a FAIRification process from netCDF files that is not explored in the literature; and ii) we have developed a new ontology for chemistry transport models by reusing vocabulary from other scientific ontologies.

## Materials and methods

**Fig. 1:**
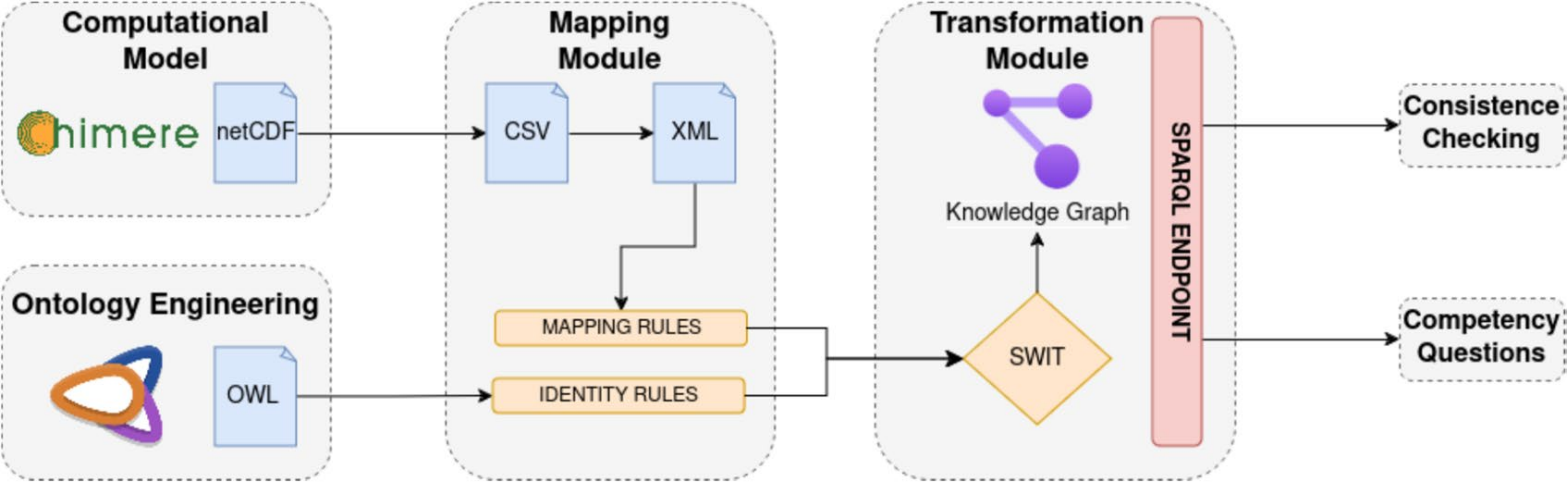
General architecture proposed for the work

Figure 1 describes the process for the generation of the knowledge graph. The process has as inputs (i) the data from the CHIMERE-WRF creating pollutant concentration simulations for the next 72 h stored in a netCDF file (Computational model); and (ii) the ontology in OWL format that provides the concepts and properties for describing the data from the computational model simulations (Ontology Engineering). First, the mapping module translates the information in the netCDF file into an XML using the *tabular2xml* library. Next, the mappings between the XML schema and the ontology are defined for SWIT, which includes two types of rules: mapping rules and identity rules. The latter ones permit merging data entities found equivalent by the application of the identity rules. Once the mapping is available, SWIT generates the semantic enriched knowledge graph with the CHIMERE data. Finally, the knowledge graph generated is evaluated by performing queries for testing the consistency of the data and the capability for answering the competency questions. In the next subsections we describe these steps.

### Computational model

The mesoscale model selected to perform the air quality forecasting is CHIMERE, an open-source multi-scale chemistry-transport model designed to produce (i) accurate analysis of pollution episodes, (ii) daily forecasts of ozone, aerosols and other pollutants, (iii) long-term simulations (entire seasons or years) for emission control scenarios. It has many different options for simulations which make it also a powerful research tool for testing parameterizations. CHIMERE is a parallel model that has been tested on machines ranging from desktop PCs running the GNU/Linux operating system, to massively parallel supercomputers [8].

**Fig. 2:**
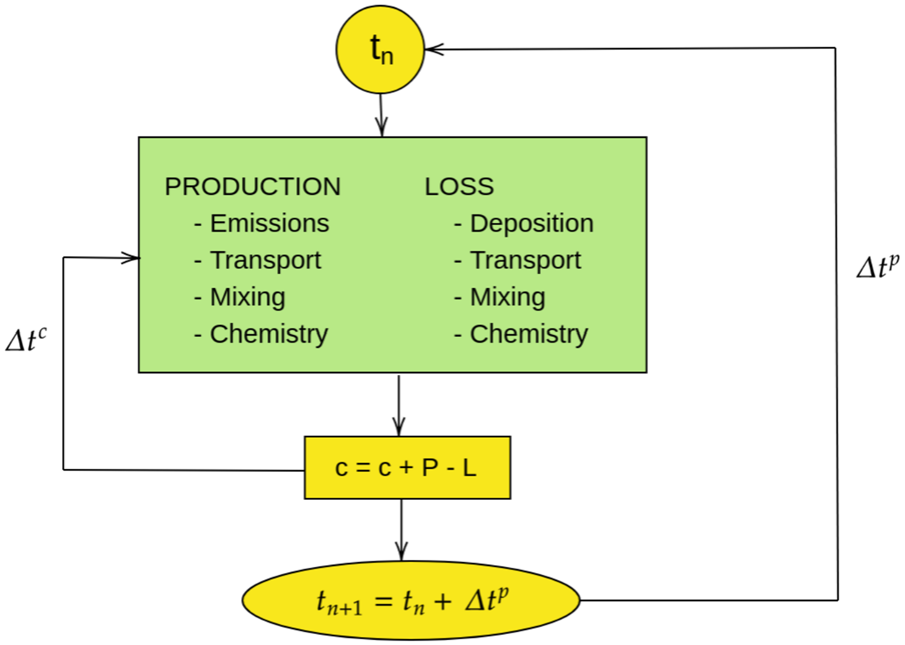
Principle of “operator-splitting” versus CHIMERE integration. Adapted from [8]

The numerical method used to estimate the temporal solution of the stiff system of partial differential equations is adapted from the second-order TWO STEP algorithm originally. It is based on the application of a Gauss–Seidel iteration scheme to the 2-step implicit backward differentiation (BDF2) formula. This means that all production and loss terms for each chemical species are calculated simultaneously to avoid error propagation generally created with operator-splitting techniques [8] (see Fig. 2).

The mesoscale simulations have been performed with the CHIMERE-WRF coupled model, in concrete version v2020r3 [38]. A gridded domain of 15 × 15 km filled by cells of 10 km^2^ has been generated. The meteorology boundary conditions have been downloaded from NCEP FNL Operational Model Global Tropospheric Analyses, continuing from July 1999 (https://rda.ucar.edu/datasets/ds083.2/dataaccess/), and the emission inventories from CAMS Copernicus Atmospheric Monitoring Service (https://eccad3.sedoo.fr/). The emission fluxes in the grid points are computed with the *emisurf* preprocessor, and the chemical kinetics have been modeled with the MELCHIOR 1 reaction mechanism [39]. The simulation period covers the whole of 2020 with 1-h timestep.

### Ontology engineering

Pollutants are small molecules products of chemical processes present in the atmosphere, as well as emissions from anthropogenic and natural sources. Concentration levels of different pollutants in the atmosphere provide evidence for which processes have taken place, and the exposure of citizens and related diseases, as well as providing additional support for sustainability impact assessment studies [40]. This approach is similar to metabolites, small molecules indicators of disorders [41]. In this sense, most of the pollutants are defined in ChEBI ontology as they have a biological effect. The main difference between metabolites and pollutants is that the first are present inside the cell while the pollutants are external agents present in the ambient, in this case the atmosphere.

The OWL ontology was created using Protégé 5.6.3, using an approach like the one proposed for metabolites, in which each forecast is a part of a whole simulation. In this case, a concentration is modeled as a separate entity that has associated a chemical entity defined in ChEBI or other vocabularies. This is a simplification since air quality forecasts concentration values may depend on how the simulation was performed and how the values were extracted and processed. The ontology was designed following the guidelines regarding human readability, in concrete about class names, descriptions and synonyms [42], by reusing terms from different ontologies, more concretely, CHEBI [34], ENVO [35] and PATO [43].

Once the ontology is defined, the next step is to evaluate it. This has been done with both the ROBOT [44] and the OQuaRE frameworks [45]. The ROBOT framework provides a series of quality control SPARQL queries over the input ontology and generates a TSV or YAML report file based on the results. Each query has a logging level to define the severity of the issue: (i) ERROR: must be fixed before releasing the ontology; (ii) WARN: should be fixed as soon as possible; and (iii) INFO: should be fixed if possible. The results of these tests have provided an overview if the ontology can be published for a formal point of view. The OQuaRE framework adapts the SQuaRE software quality standard and provides the following quality characteristics for ontologies: reliability, operability, maintainability, compatibility, transferability and functional adequacy. 19 metrics are used for measuring such quality aspects and assign a score between 1 (lowest)−5 (highest) to the metrics and to the quality categories. In this sense, Table 1 describes the metrics provided by OQuaRE [45]. One of the strengths of the method is that these metrics could be used to compute clustering and comparison with other ontologies in the state of the art by using the *evaluomeR* framework [46].

**Table 1:** OQuaRE quality metrics definition

Metric	Definition
ANOnto	Mean number of annotations per class
AROnto	Mean number of attributes per class
CBOOnto	Number of related classes. It is the average number of the direct parents per class minus the relationships of Thing
CBOnto2	Same as CBOOnto, it changes the computation method
CROnto	Mean number of instances per class
DITOnto	Max (∑D|C_i_|), where C_i_ are the classes and D|C_i_| is the length of the path from the i leaf class of the ontology to Thing
INROnto	Mean number of relationships per class
LCOMOnto	The semantic and conceptual relatedness of classes can be used to measure the separation of responsibilities and independence of components of ontologies
NACOnto	Mean number of ancestor classes per leaf class. It is the number of direct superclasses per leaf class
NOCOnto	Mean number of direct subclasses. It is the number of relationships divided by the number of classes minus the relationships of Thing
NOMOnto	Number of properties per class
RFCOnto	Number of properties that can be directly accessed from the class
PROnto	Number of subclass of relationships divided by the number of subclass of relationships and properties
TMOnto	Mean number of parents per class
TMOnto2	Same as TMOnto, it changes the computation method
POnto	Mean number of direct ancestors per class
RROnto	Number of properties defined in the ontology divided by the number of relationships and properties
WMCOnto	Mean number of properties and relationships per class
WMCOnto2	Same as WMCOnto, it changes the computation method

### Knowledge graph generation

The knowledge graph generation starts from a *netCDF* file and ends in an RDF file. This process is composed of three steps. (i) the transformation of *netCDF* in a *csv* file; (ii) the transformation of the tabular file into an XML file; and (iii) the mapping of the XML file to the ontology developed, so the netCDF data can be represented in RDF.

The first step was performed using the *netCDF* Python package [47]. This allowed us to extract the different variables over the dimensions and locate them in the csv. During this step, three preprocessing steps were applied: The first one was the selection of the data to be included in the knowledge graph. The second step is the generation of the XML file, which has been done using the *tabular2xml* file. This creates an XML in which each entity corresponds to a file in the *csv.* Also, the software generates the XSD file with the schema configured.

**Fig. 3:**
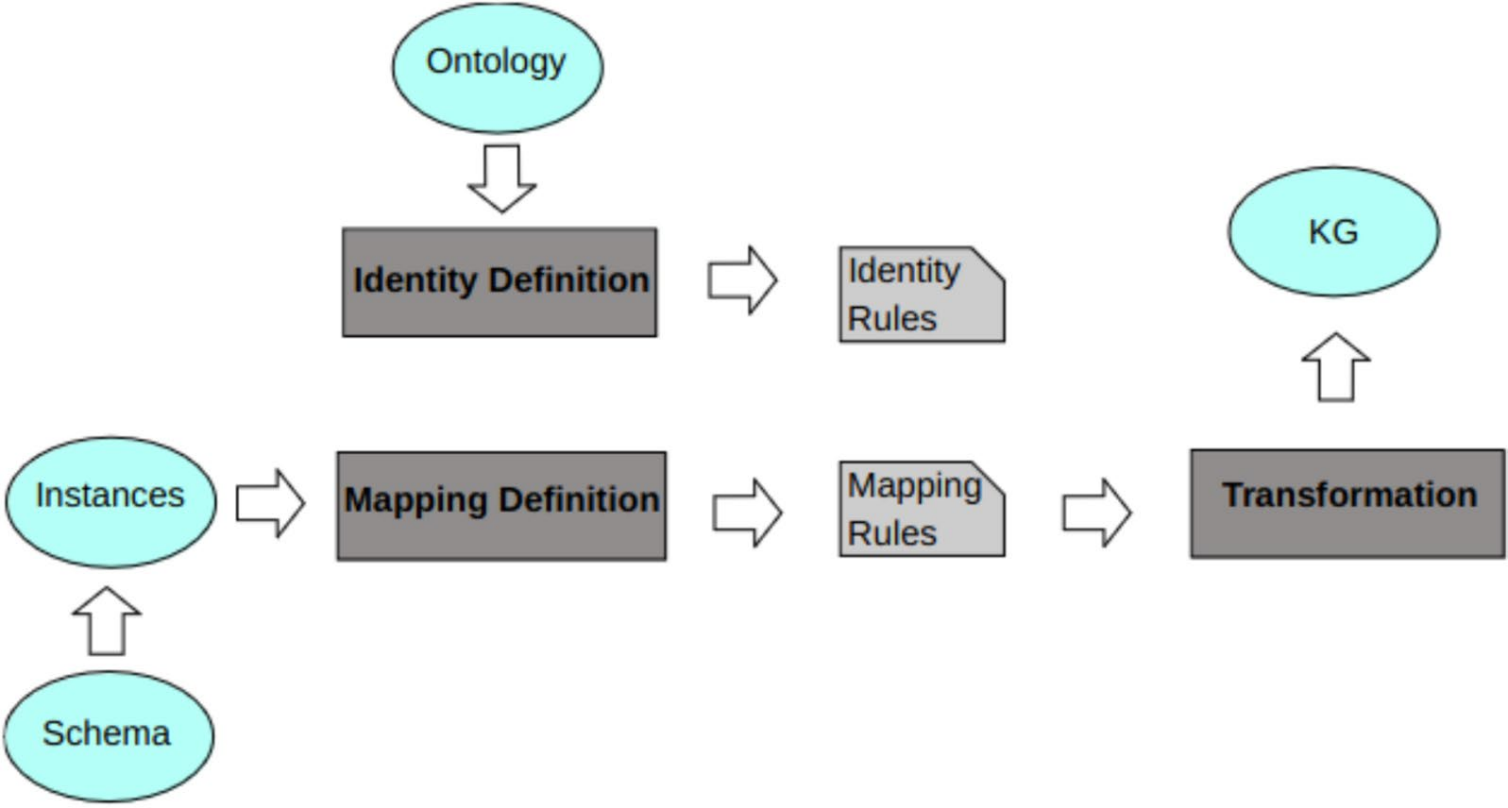
SWIT architecture. The framework requires the input instances, a mapping rules file and an ontology. Adapted from [32]

Last, the key part of the process is the generation of the knowledge graph. This is done using the SWIT framework, which transforms XML data into repositories in Semantic Web formats. SWIT provides a semantics-rich, ontology-driven transformation and integration of datasets. SWIT repositories are not redundant and are logically consistent with the axioms of the ontology used in the process [13]. It requires an input dataset, the input schema, an OWL ontology and one user-defined transformation file containing the mapping rules, which define how the entities of the input schema are mapped to the ontology [37]. Figure 3 shows the SWIT functional architecture.

In concrete, mapping rules guarantee that the information represented according to the input schema is correctly transformed [37]. In this case, we defined (i) entity rules to create individuals of the CHIMERE ontology; (ii) attribute rules to define the concentration values for concentration individuals—and the same for physical parameters; and (iii) relation rules to link concentration individuals with chemical entities and other relationships between individuals. The knowledge graph generation process is summarized in *Algorithm 1,* creating knowledge graph representations for each entity using the SWIT tool, and appending the resulting triples to the final RDF file. In this algorithm, the functions *createRDF()* and *createRoot()* initialize an empty RDF graph and an empty XML root. Over this RDF graph, the algorithm appends new triples in each iteration while *createRoot()* is used to define an auxiliary variable with a temporal XML. This is done with the function *createTemporalXML(root)* that takes as parameter a *root*. Last, the function *append(graph1, graph2)* appends a set of RDF triples into another RDF graph.

**Algorithm 1:**
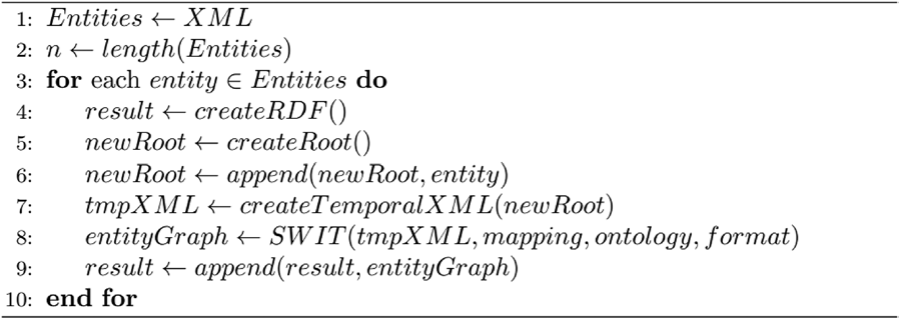
Knowledge graph generation from an XML input, creating knowledge graph representations for each entity using the SWIT tool, and appending the resulting entity graphs to RDF

### Consistency checking

Once the triples are generated, it is important to determine if they are consistent with the semantics and requirements defined. This is done through the temporal consistency and spatial consistency queries, written in SPARQL, which can be consulted in the following link: https://github.com/eduardoillueca/chimere-ontology/tree/main/SPARQL. The spatial consistency query retrieves information related to air quality forecasts from a dataset using the CHIMERE ontology, assessing that there is one air quality forecast instance for each timestamp forecasted. The query selects all instances where a forecast is associated with a municipality and a specific timestamp. The triple patterns "?forecast hasMunicipality ?municipality" and "?forecast hasTimestamp ?date"establish connections between the forecast, its associated municipality, and the timestamp. The FILTER clause further refines the results by specifying a condition that the timestamp (?date) must equal the value "1702965600000" with the datatype xsd:decimal. In essence, this query seeks to identify and retrieve air quality forecast data for a specific municipality at a precise timestamp, allowing for a focused examination of spatial consistency in air quality predictions for the specified time and location within the CHIMERE ontology dataset.

**Query 1:**
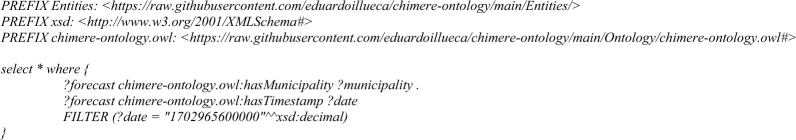
Spatial consistency query

On the other hand, the temporal consistency query is designed to extract information from a dataset utilizing the Chimere ontology, focusing on air quality forecasts. The query selects instances where a forecast is associated with a municipality and captures the corresponding timestamp. The triple patterns *?forecast hasMunicipality? municipality* and ?*forecast hasTimestamp?date* establishes the link between the forecast, its associated municipality, and the timestamp. The FILTER clause refines the results by specifying a condition that the municipality (?municipality) must equal the literal value"Totana". Essentially, this query seeks to retrieve air quality forecast data specifically for the municipality of Totana, emphasizing a temporal consistency analysis for air quality predictions related to Totana within the CHIMERE ontology dataset.

**Query 2:**
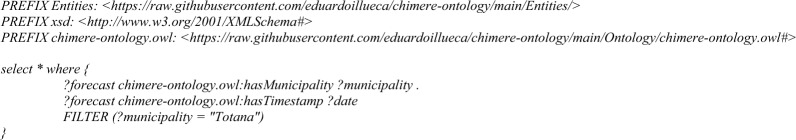
Temporal consistency query

### Competency questions

Finally, the knowledge graph has been evaluated through a series of competency questions, represented as four different SPARQL queries that can be found in the repository https://github.com/eduardoillueca/chimere-ontology/tree/main/SPARQL. These competency questions were carefully designed to ensure they comprehensively evaluate the knowledge graph’s ability to answer key domain-specific queries. It is important to note that this approach is for evaluating the transformation from netCDF, so this information can also be extracted from the source netCDF file, and there is no reasoning at this point. By proceeding in this way, we can test the correctness of the mapping done. To achieve this, we first identified typical use cases for air quality monitoring and management, including time series extraction for a location, plotting heatmaps at a regional scale, identifying threshold superations and computing the air quality index.

–**Time Series Extraction**: this query seeks to obtain the time series for a specific municipality and then plot it. The query selects two variables, ?date and ?value, representing the timestamp and concentration value of a particular chemical entity. The query starts by identifying forecasts associated with the municipality and extracts the timestamp for each forecasted event. This query is like the temporal consistency test, but it is necessary to specify the chemical entity to extract from the knowledge graph. In this example (*Query 3*), the PM10 pollutant is the target and, in the case to analyze a CHEBI pollutant, the CHEBI identifier should be put as the second parameter of REGEX.

**Query 3:**
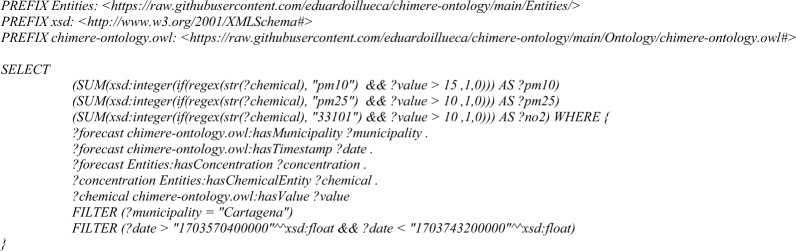
Time series competence query

–**Spatial Heatmap**: like the previous one, this test seeks to extract the data for all the municipalities in a concrete timestamp and plot it into a heatmap. This is done through *Query 4*, following the same philosophy as the spatial consistency but filtering for chemical entities. This query is useful for extracting data related to air quality or environmental monitoring, where concentrations of specific chemicals in different municipalities at a particular timestamp are of interest.

**Query 4:**
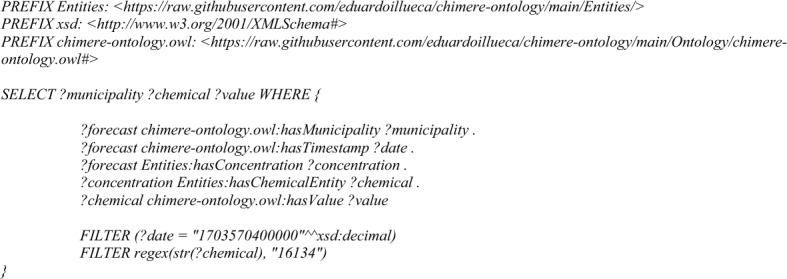
Spatial heatmap competence query

–**Counting of the number of superations in a time range**: this query will identify the number of superations for each pollutant in the next 24 h. A superation is a timestamp in which the level of a certain pollutant reaches or exceeds a concentration threshold, which is defined by an air quality expert and can be a regulatory limit, a concentration that is harmful from health or other indicator that is relevant for the use case. In this competency question, the limit values to be used could be the ones defined by the World Health Organization for 24 h [48], but for practical reasons has been fixed to 10 μg/m^3^ form PM10 and NO_2_, and 5 μg/m^3^ for PM2.5.

**Query 5:**
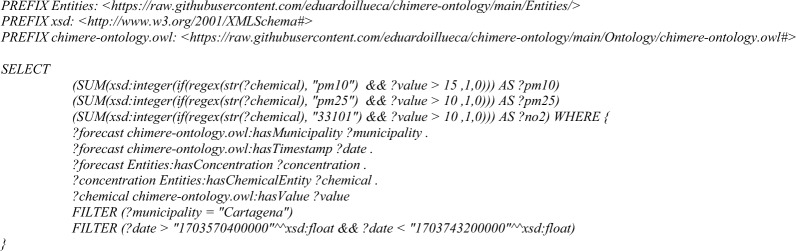
Number of superations competence query

–**Calculation of the Air Quality Index (AQI) for a concrete timestamp**: Concentration values for up to five key pollutants determine the index level that reflects air quality at each monitoring station [49]. The index corresponds to the poorest level for any of five pollutants, according to Table 2, labeled with the colors indicated by the European Commission.

**Table 2:** European air quality index

Pollutant	Good	Fair	Moderate	Poor	Very Poor	Extremely Poor
PM2.5	0–10 μg/m^3^	10–20 μg/m^3^	20–25 μg/m^3^	25–50 μg/m^3^	50–75 μg/m^3^	75–800 μg/m^3^
PM10	0–20 μg/m^3^	20–40 μg/m^3^	40–50 μg/m^3^	50–100 μg/m^3^	100–150 μg/m^3^	150–1200 μg/m^3^
NO_2_	0–40 μg/m^3^	40–90 μg/m^3^	90–120 μg/m^3^	120–230 μg/m^3^	230–340 μg/m^3^	340–1000 μg/m^3^
O_3_	0–50 μg/m^3^	50–100 μg/m^3^	100–130 μg/m^3^	130–240 μg/m^3^	240–380 μg/m^3^	380–800 μg/m^3^
SO_2_	0–100 μg/m^3^	100–200 μg/m^3^	200–350 μg/m^3^	350–500 μg/m^3^	500–750 μg/m^3^	750–1200 μg/m^3^

For testing this functionality, *Query 6* is designed to calculate the Air Quality Index (AQI) for a concrete municipality, based on the concentration values of different air pollutants measured during a specified timestamp range.

**Query 6:**
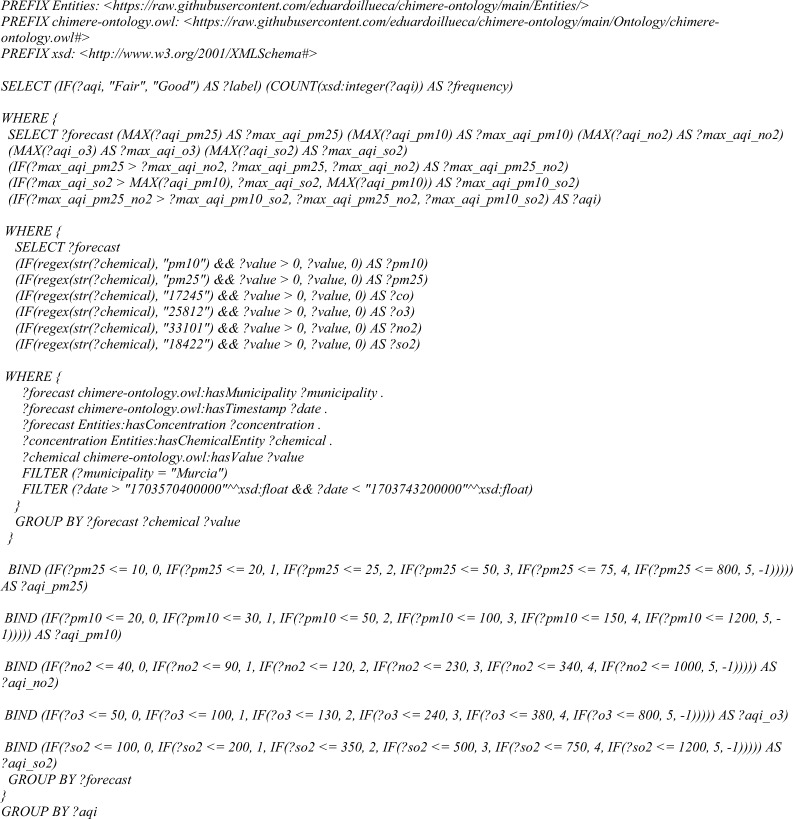
AQI competence query

## Results

The results section describes the ontology developed and the knowledge graph obtained for our use case.

### The ontology

**Fig. 4:**
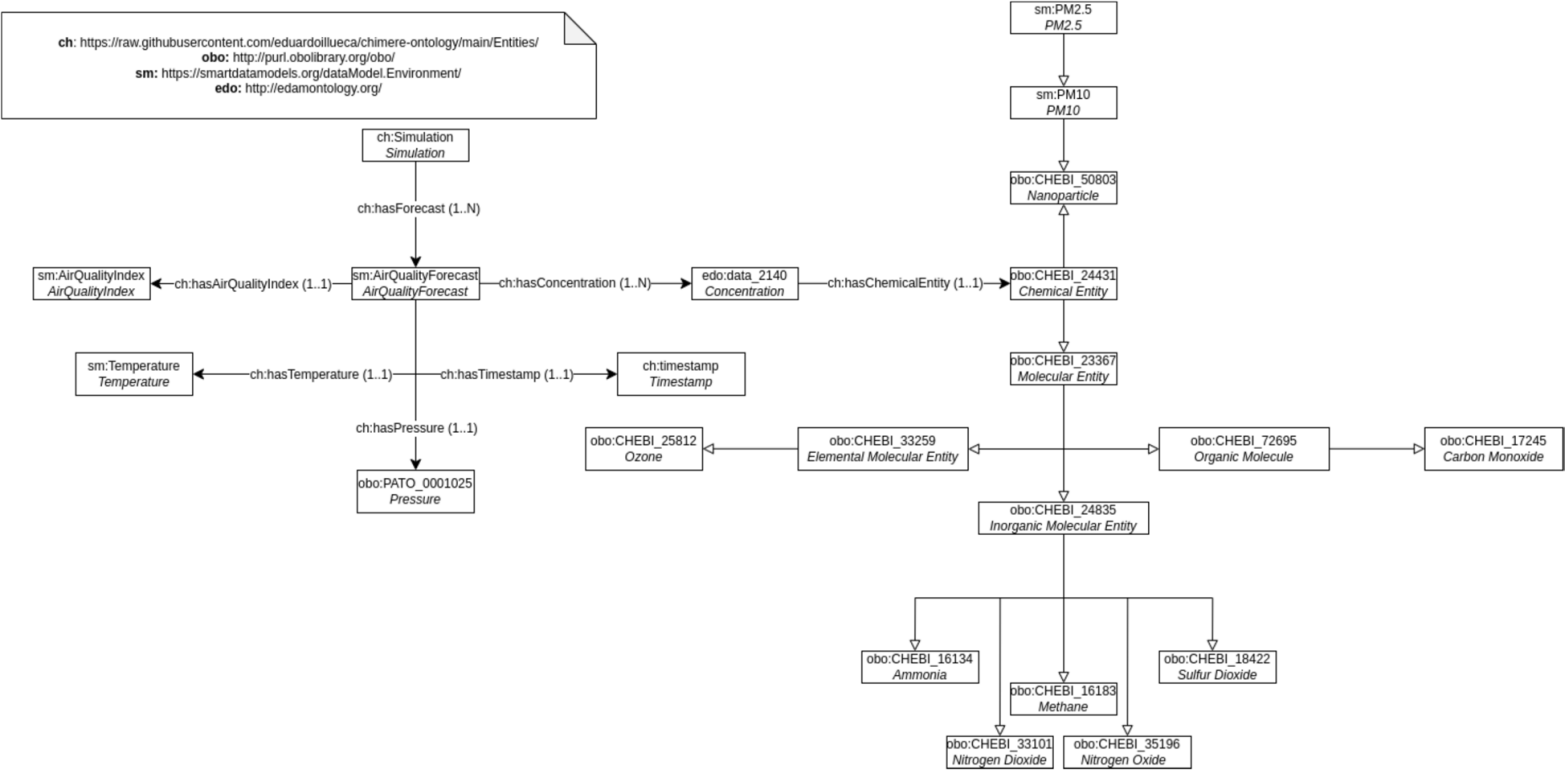
Conceptual design of the CHIMERE ontology. Unlabeled arrows represent the property *subClassOf*. The label of each class is shown in italics

The first step in ontology assessment is to describe the conceptual design after following the methodology described in *Materials and Methods*. This representation is shown in Fig. 4. The core of ontology is the concept of *Simulation*, which refers to the result of a running of CHIMERE. Each simulation is composed of a set of instances of *Air Quality Forecast*, which is the part of the simulation linked to a geospatial unit and a timestamp, and it is possible to infer an *Air Quality Index*. Each air quality instance can have several *Concentration* instances, which are linked to a unique *Chemical Entity*, which can be of one of the different subclasses presented in Fig. 4. The ontology can be downloaded in OWL format from PURL (https://purl.org/chimere-ontology) and it is available in the following repository: https://github.com/eduardoillueca/chimere-ontology.

Regarding the syntactic and formal validation, a total of 31 tests have been done through the ROBOT report interface. As a summary, it is important to highlight that all the tests were passed successfully, indicating that the ontology is consistent in terms of formulations and syntax. Next, we present the results of the application of the 19 metrics of the OQuaRE framework (see Table 3). There, each metric has a score ranging from 1 to 5 assigned, where *1 means not acceptable, 3 is minimally acceptable, and 5 exceeds the requirements*. In general, all the metrics are acceptable with high scores (4–5), except CROnto metric and the RROnto metric. According to Franco et al. [50], the CROnto metric increases with the number of individuals. Thus, the score 1 is obtained because the ontology is not populated with individuals, as the idea is to use it as a template to create individuals in the output knowledge graphs. On the other hand, the result on RROnto shows a lack of usage of the data properties and object properties inside the ontology, and this issue should be reviewed in future versions of the ontology.

**Table 3:** Oquare results

Metric	Range	Score
ANOnto	> 80%	5
AROnto	(60–80] %	4
CBOOnto	[1,2]	5
CBOnto2	[1,2]	5
CROnto	[0–20] %	1
DITOnto	(4,6]	3
INROnto	(60–80] %	5
LCOMOnto	(2–4]	4
NACOnto	[1,2]	5
NOCOnto	[1,3]	5
NOMOnto	$\le$ 2	5
RFCOnto	[1,3]	5
PROnto	–	4
TMOnto	(1,3]	5
TMOnto2	(1,3]	5
POnto	–	5
RROnto	(20–40] %	2
WMCOnto	$\le$ 2	5
WMCOnto2	$\le$ 2	5

**Fig. 5:**
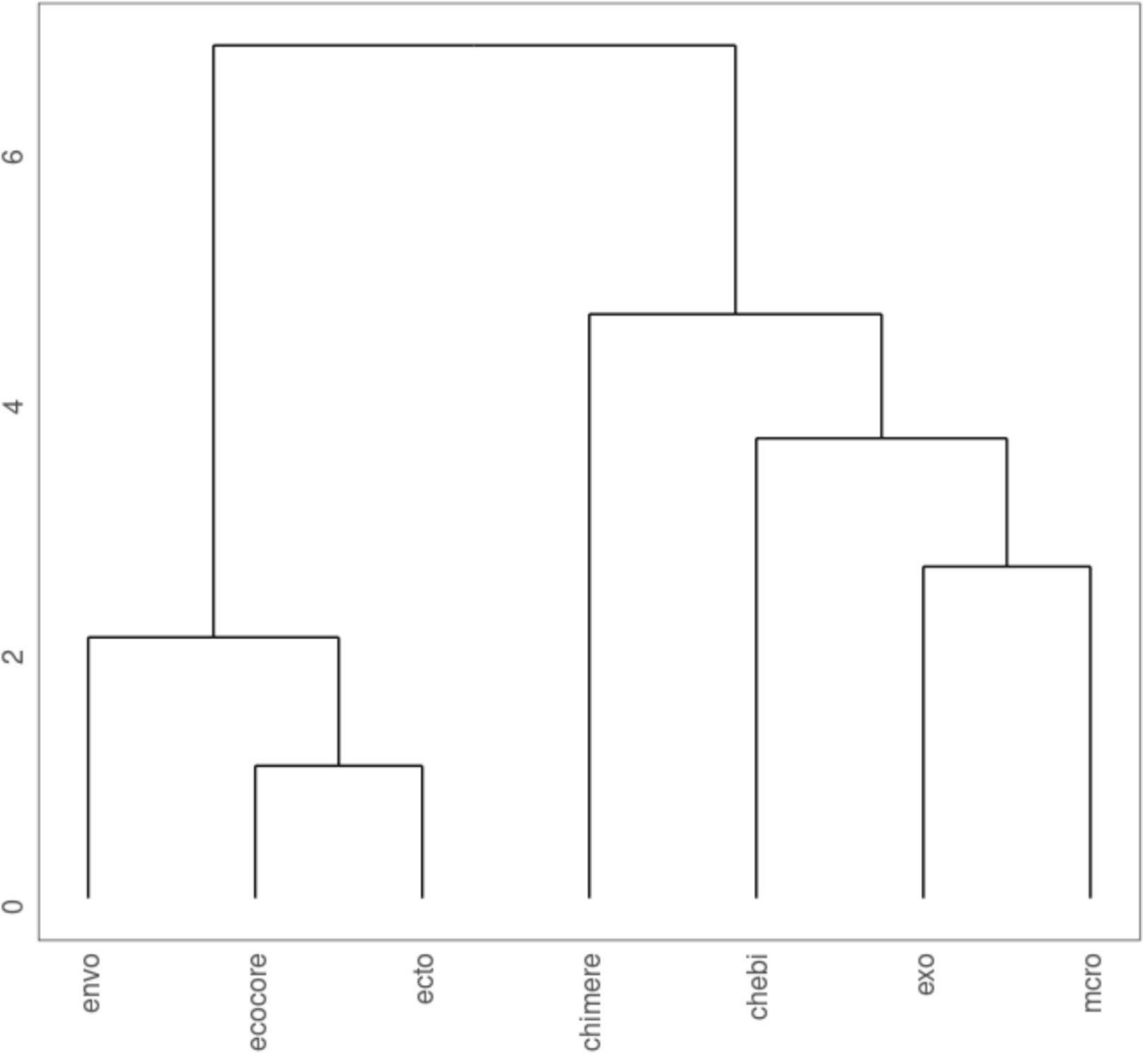
Comparison of CHIMERE ontology with other ontologies in state of the art, according to the metrics computed by OQuaRE

Next, we compare the OQuaRE results of our ontology with the results of related ontologies as ENVO [35], ECTO [51], CHEBI [34], EXO [52], MCRO [53] and ECOCORE [54] by using the *EvaluomeR* package. In this case, we have carried out a hierarchical clustering according to the *ward* method, resulting in the dendrogram presented in Fig. 5. The clustering has two main branches. The first one represents the *environmental* ontologies and the second one *chemical* and *mathematical* ontologies. The CHIMERE ontology is in the chemical and mathematical branch, being the CHEBI ontology the closest one.

### The knowledge graph

#### Description of the use case

The Region of Murcia is an administrative region in the south of Spain, whose capital resides in the city of Murcia. The economy of Murcia is mainly based in agriculture but also has important industry zones in the area of Molina de Segura and Cartagena. For this reason, air quality monitoring and management is one of the key challenges for public administrations in Murcia. However, the current air quality network only has eight monitoring points that does not cover all the municipalities in the region. For this reason, there is a need to obtain data and indicators for these municipalities by using chemistry transport models and it is also important that this information should be interoperable with other administrations and systems.

To address these challenges, we have applied our ontology-driven approach to generate semantically enriched data, thus enabling better integration and interoperability of air quality information. The CHIMERE-WRF model has been used to generate data for each one of the municipalities in the region. In addition, we provide an infrastructure for storing the knowledge graphs generated and provide an endpoint to consult the data. All these components are described in the section Materials and Methods.

The actors involved in this use case are the public administration responsible for ensuring air quality data for the environmental monitoring system and making policy decisions. Also, citizens can also benefit from the data generated. The main flow is divided into four steps: (i) air quality forecasting with CHIMERE, (ii) CSV and XML generation, (iii) knowledge graph generation and (iv) data storing and querying.

The database selected to store the knowledge graphs generated with SWIT is GraphDB, a semantic repository, packaged as a Storage and Inference Layer (Sail) for the RDF4J framework and it makes extensive use of the features and infrastructure of RDF4J, especially the RDF model, RDF parsers, and query engines. Thus, GraphDB provides a standard SPARQL 1.1 endpoint so it is fully interoperable with any SPARQL 1.1 client. In addition, the SPARQL protocol for RDF allows transmitting SPARQL queries to a SPARQL query processing service and returning the query results to the entity that requested them. One of the strengths of GraphDB is the federation of repositories, allowing it to speed up the queries [55].

#### Description of the data sources

CHIMERE computes a wide range of chemical and physical parameters, but not all them were relevant for the proposed use case. For this reason, only NO_2_, NO, O_3_, CO, SO_2_, CH_4_, NH_3_, temperature and pressure are selected, helping to reduce the computational cost. The second assumption relies on dimensionality reduction. CHIMERE is a 3D model, allowing to compute concentration over different heights. However, in our use case, the only level of relevance is the surface level, thus one dimension was removed from the netCDF file, reducing the computational cost. Last, CHIMERE concentrations are calculated over gridded domains, but a concentration for a cell has no sense for policy makers. Thus, cell values are interpolated over the 45 municipalities in the Region of Murcia. This is done by computing the surface pondered mean of all the grids inside a municipality. To perform the simulations, CHIMERE needs to integrate different physico-chemical data sources that are summarized in Table 4.

**Table 4:** Use case data sources

Data source	Description
Copernicus atmosphere monitoring service	This dataset involving inventory was developed for the years 2000–2023 for the atmospheric compounds included in the CAMS model. Emissions are provided for 17 sectors, depending on the species. The spatial resolution of the inventory is 0.1 × 0.1 degree. Emissions are provided as monthly averages. The dataset is based on the EDGARv5 annual emissions to which we apply the monthly temporal profiles from CAMS-GLOB-TEMPO. The emissions are extended to the most recent years using trends from the CEDS global inventory Available: https://eccad.sedoo.fr/#/catalogue
NCEP FNL operational model global tropospheric analyses	These NCEP FNL (Final) Operational Global Analysis data are on 1-degree by 1-degree grids prepared operationally every six hours. This product is from the Global Data Assimilation System (GDAS), which continuously collects observational data from the Global Telecommunications System (GTS), and other sources, for many analyses. The FNLs are made with the same model which NCEP uses in the Global Forecast System (GFS), but the FNLs are prepared about an hour or so after the GFS is initialized. The FNLs are delayed so that more observational data can be used. The GFS is run earlier in support of time critical forecast needs and uses the FNL from the previous 6-h cycle as part of its initialization Available: https://rda.ucar.edu/datasets/ds083.2/dataaccess
MELCHIOR Reaction Mechanism	This dataset is a reaction mechanism including the main chemical reactions that occur in the atmosphere as well as the kinetic constant to model each reaction Available: https://www.lmd.polytechnique.fr/chimere/

#### RDF generation with SWIT

The knowledge graph generated by SWIT comprises a simulation of 73 h, with a total of 154,484 individuals and 266,369 triples, implying a file size of 56.1 MB in the RDF/XML format. All these statistics are summarized in Table 5.

**Table 5:** Knowledge graph statistics

Parameter	Value
Number of simulated hours	73 h
Total number of individuals	154,484 individuals
Number of triples	266,369 triplets
Size (RDF/XML)	56.1 MB

Table 6 provides more details on how the knowledge graph is populated, calculated using the SPARQL endpoint. As the simulation comprises 73 h for 45 municipalities, the total number of individuals for the *AirQualityForecast* class is 73 × 45 = 3285, showing consistency with the definition done for *AirQualityForecast* in Fig. 4. The rest of classes present the logical number of individuals according to the cardinality defined in the ontology.

**Table 6:** Individuals per class

Parameters	Number of individuals
Air Quality Forecast	3285
Concentration	29,565
Temperature	3285
Pressure	3285
Chemical Entity	29,565
Elemental Molecular Entity	3285
Organic Entity	3285
Inorganic Entity	16,425
NO_2_	3285
NO	3285
O_3_	3285
SO_2_	3285
CH_4_	3285
NH_3_	3285
CO	3285
Nanoparticle	6570
PM10	6570
PM2.5	3285

**Fig. 6:**
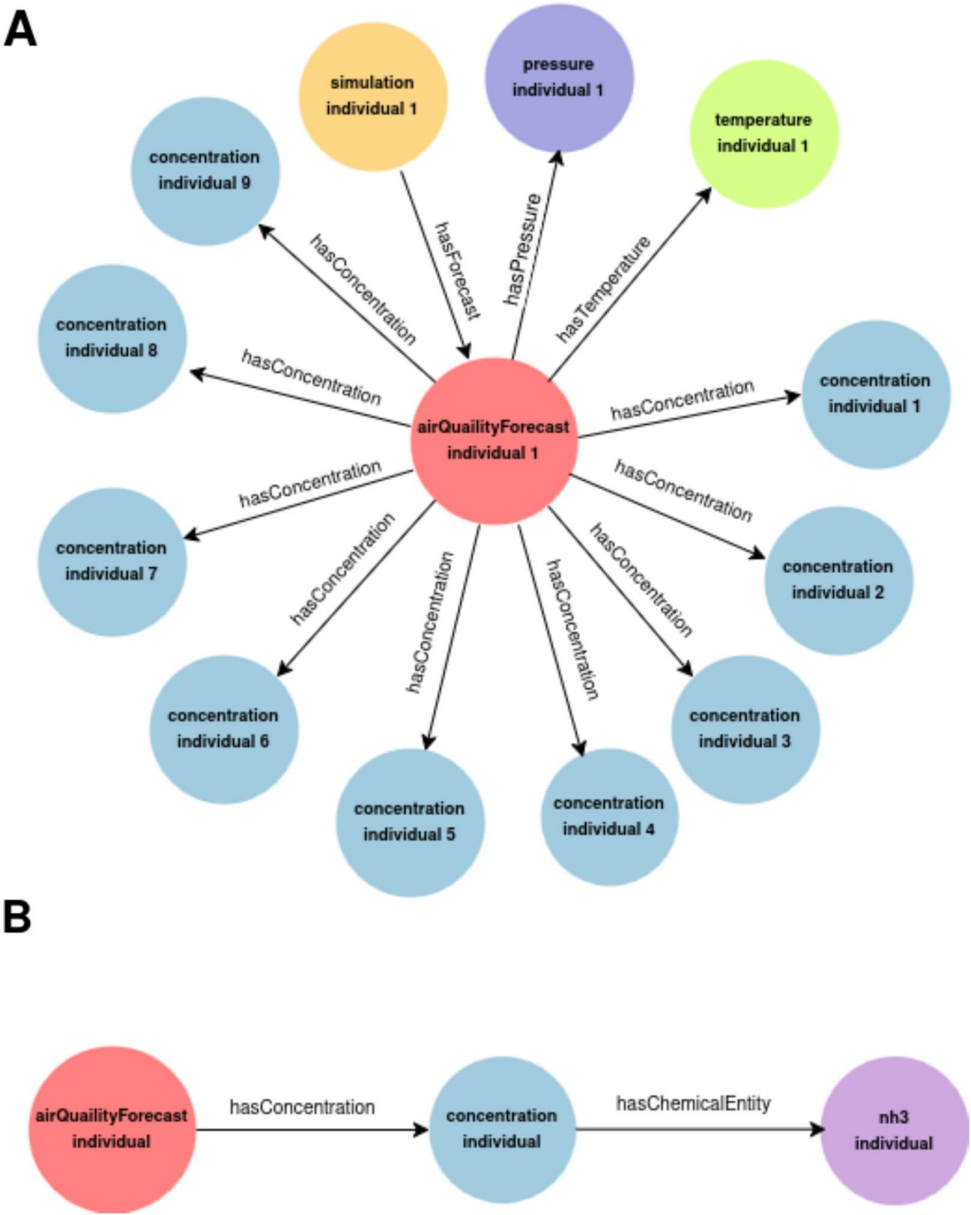
Knowledge graph structure. **A** The different relationships of an AirQualityForecast individual with nine concentration individuals through the object property hasConcentration; **B** The relationship of a Concentration individual with an nh3 individual

Figure 6 provides more detail in these results by providing insights regarding the structure of the knowledge graph. Figure 6A shows an *AirQualityForecast* individual and its relationships. Each air quality individual is linked with nine concentration individuals through the object property *hasConcentration*, and with one temperature individual and one pressure individual*.* Figure 6B shows the link between a Concentration individual and a *nh3* individual. According to the range of the object properties, an *AirQualityForecast* individual has only one *Temperature* and only one *Pressure*. Thus, the number of individuals belonging to *Temperature* and *Pressure* is 3825. Regarding *Concentration*, for this simulation, each *AirQualityForecast* is linked with 9 *Concentration* individuals, making the total number of individuals belonging to this class 3285 × 9 = 29,565. The bottom part shows that a *Concentration individual* which is related through the *hasChemicalEntity* data property with only one chemical compound. For this reason, the number of individuals for each one of the chemical species is 3285, except for PM10 as, according to Fig. 4, PM2.5 is a subclass of PM10. In consequence, all the PM2.5 individuals are PM10 individuals, making a total of 6570 belonging to this class.

#### Performance of the knowledge graph generation process

**Fig. 7:**
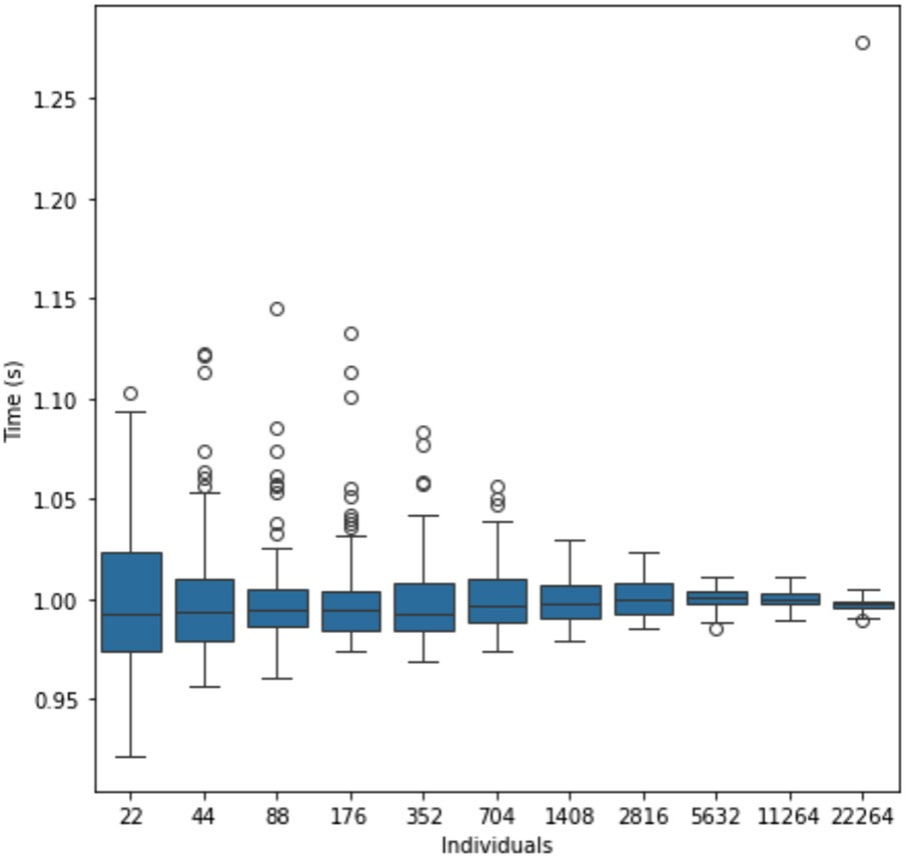
Boxplots showing the normalized time distribution when incrementing the number of AirQualityForecast individuals

The computational performance of SWIT has been tested in 100 different tests, in which a sample dataset of 2^n^ forecast hours have been selected, ranging from n = 0 to n = 10. The first result to be noted is that for each hourly forecast the number of individuals increase by 22, taking the following growing formulas $i = 22h$*,* where i is the number of *AirQualityForecast* individuals and h the number of hours to be forecasted. From a qualitative point of view, it can be observed that by duplicating the number of individuals, the time spent is also duplicated, suggesting a linear complexity model. However, it is important to assess the variability of the time among the datasets to understand the computational complexity. This is done in Fig. 7*,* where it can be observed that the variability decreases when the number of individuals increases. To avoid scale problems, the time in the y-axis is normalized. This result shows that the stability of the time is greater in bigger datasets. This is logical because small time processing is affected more by I/O processes than the mapping and the SWIT computation.

**Fig. 8:**
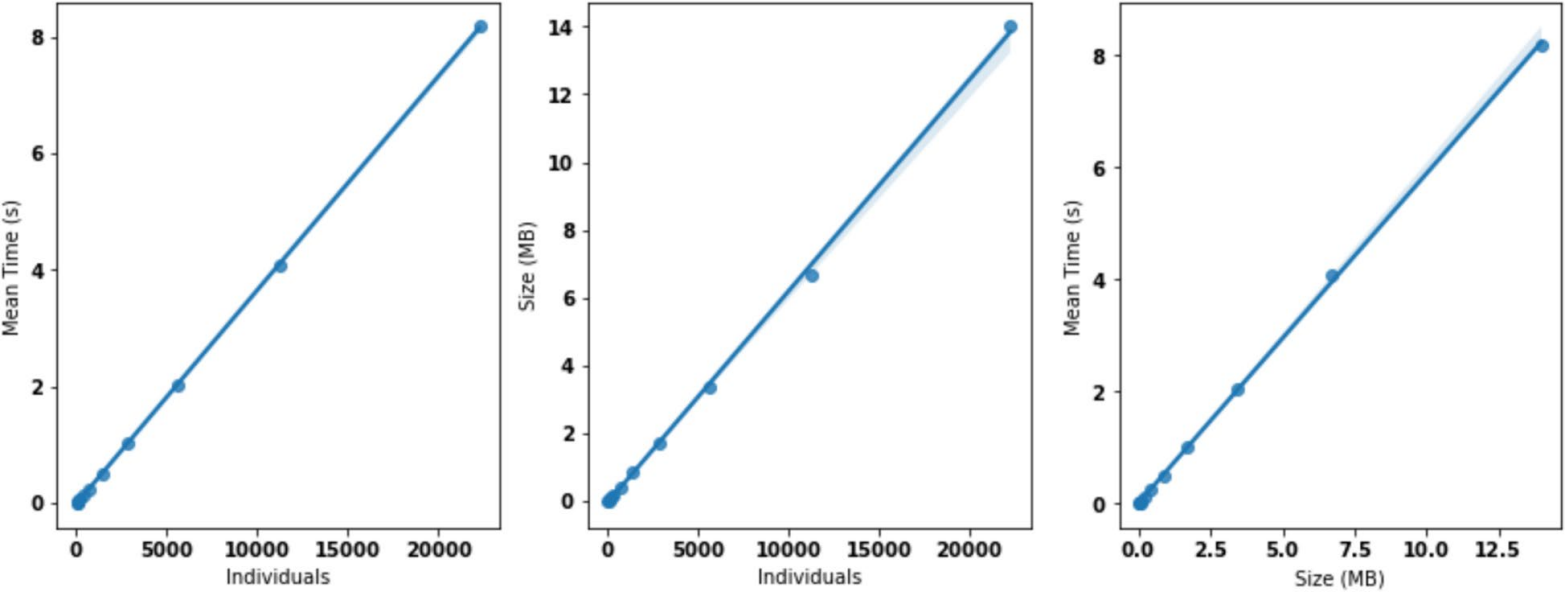
Relationship between computation mean time, size of the knowledge graph and number of AirQualityForecast individuals. In **a** we represent the mean computation time versus the number of individuals, in **b** the graph size versus the number of individuals and in **c** the mean computation time versus the graph size

The quantitative assessment is shown in Fig. 8*,* where three different models have been tested, showing all of them a linear tendency. The first one is the evolution of the time regarding the number of *AirQualityForecast* individuals, the second one is the size of the knowledge graph versus the number of individuals again and the last one is the computation time vs the size of the knowledge graph. According to the results, the computational complexity of the SWIT tool is characterized by its linear scalability, indicating that the resource requirements and processing time exhibit a proportional relationship with the size of the input. This implies that as the complexity of the workflows or the volume of data increases, the SWIT tool demonstrates a consistent, linear growth in computational demands without significant spikes or exponential surges.

The statistical analysis of the regression models proposed are presented in Table 7. The three models are significant from a statistical point of view, with R^2^ > 0.99 and a P_Value_ close to zero in the three cases.

**Table 7:** Computational performance regression results

Model	Complexity	R^2^	P-value
Time vs Individuals	O(n)	1.000	2.67e-23
Size vs Individuals	O(n)	0.999	8.09e-18
Time vs Size	O(n)	1.000	3.17e-19

#### Data consistency checking

**Fig. 9:**
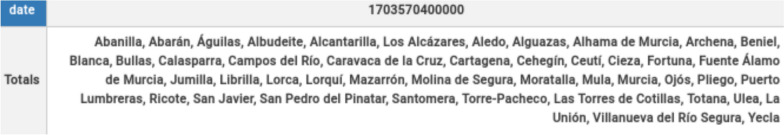
Spatial consistency result

The results of the temporal consistency check if for each date exists one *hasMunicipality* property with each one of the municipalities of the Region of Murcia. In total, there must be 45 different municipalities with no duplicates. This can be checked by applying *Query 1*. In Fig. 9, an example is shown by fixing the date to the timestamp 1703570400000, which corresponds to the date 2023-12-26 06:00:00 UTC according to the *currentMillis* standard. It is observed that the date is linked with 45 different municipalities. This test has been repeated for each one of the timestamps present in the database, obtaining the same result and proving the temporal consistency.

**Fig. 10:**
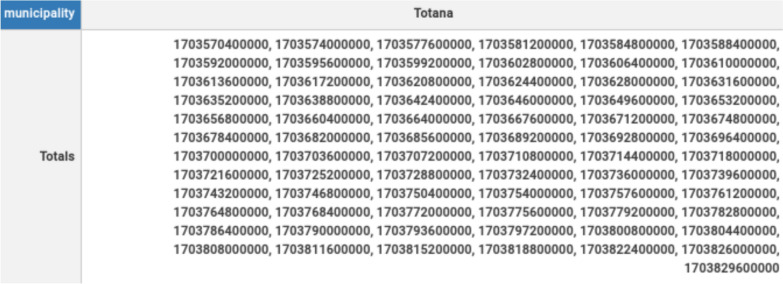
Temporal consistency result

On the other hand, the temporal consistency checks if for each municipality exists one *hasDate* linking each one of the municipalities of the Region of Murcia with different dates without duplicates. In this case, the total number of dates per municipality can change depending on the size of the database or the length of the simulation. Thus, the unique restriction is that a municipality cannot have duplicated dates. This can be assessed by applying *Query 2*. In Fig. 10, an example is shown by fixing the municipality to Totana, in the southwest of the Region of Murcia, for a simulation of 73 h. It is observed that the municipality is linked with 73 different timestamps. This test has been repeated for each one of the municipalities, obtaining the same result and proving the spatial consistency.

Last, a consistency checking is done over the output knowledge graph by using Hermit, a popular reasoner and ontology processor, employs advanced reasoning techniques to analyze and validate the logical coherence of the graph. When the knowledge graph successfully passes the consistency checking with Hermit, it indicates that the graph adheres to the defined constraints and axioms without encountering any logical contradictions or inconsistencies. In this case, the RDF/XML outputs passed successfully the consistency checking, highlighting the confidence in the accuracy of the information.

#### Evaluation through competency questions

–Time series extraction

**Fig. 11:**
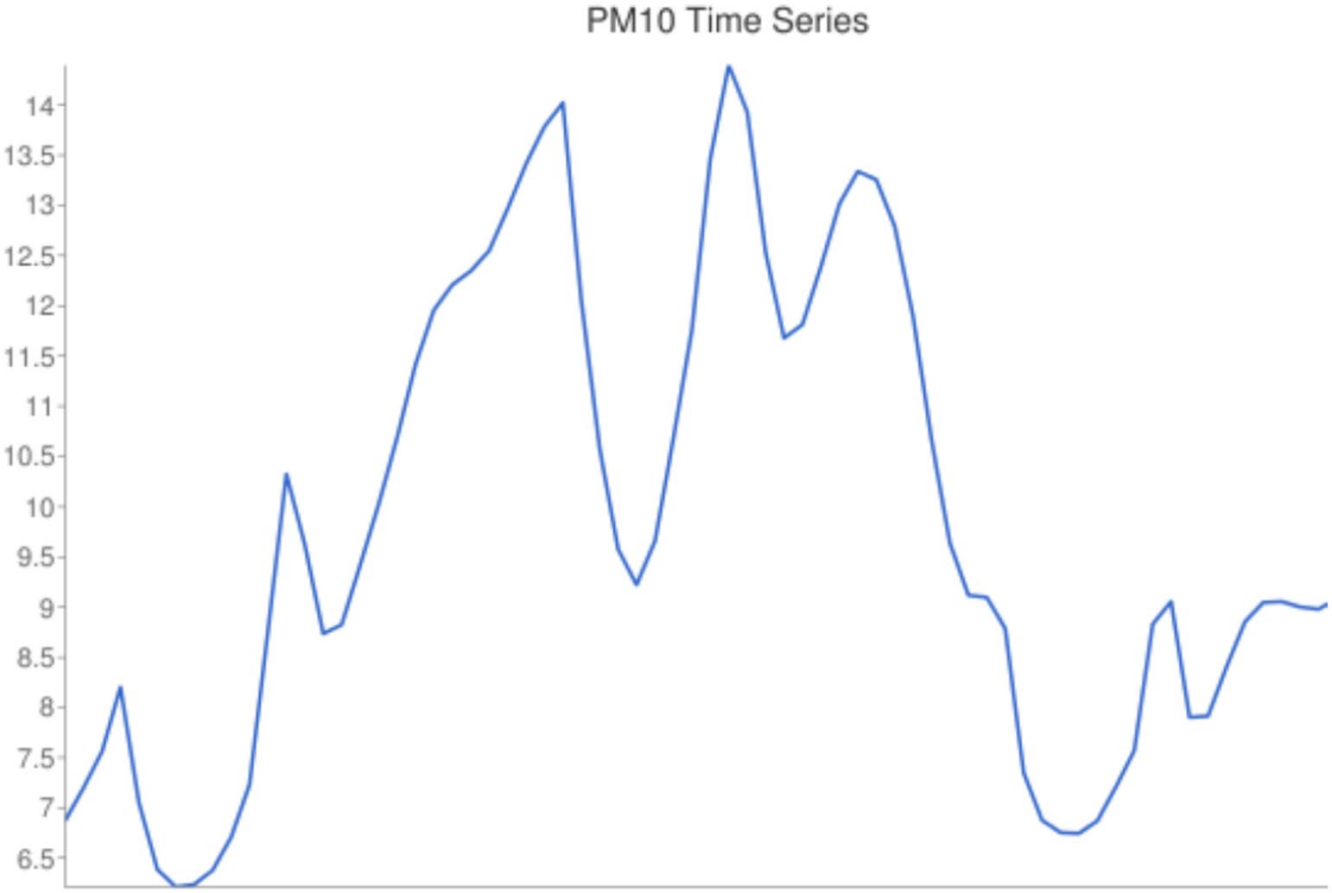
Result of the time series query, showing the evolution of PM10 in μg/m^3^ under 72 h in the municipality of Totana

The result of the first query provides a time series for a concrete pollutant, which is represented in Fig. 11. In this example, the 72 h for PM10 in the municipality of Totana is plotted, in μg/m^3^. The concentration values are between 6 and 15 μg/m^3^, and the normal tendency in particles is observed with peaks and valleys.

–Spatial heatmap

**Fig. 12:**
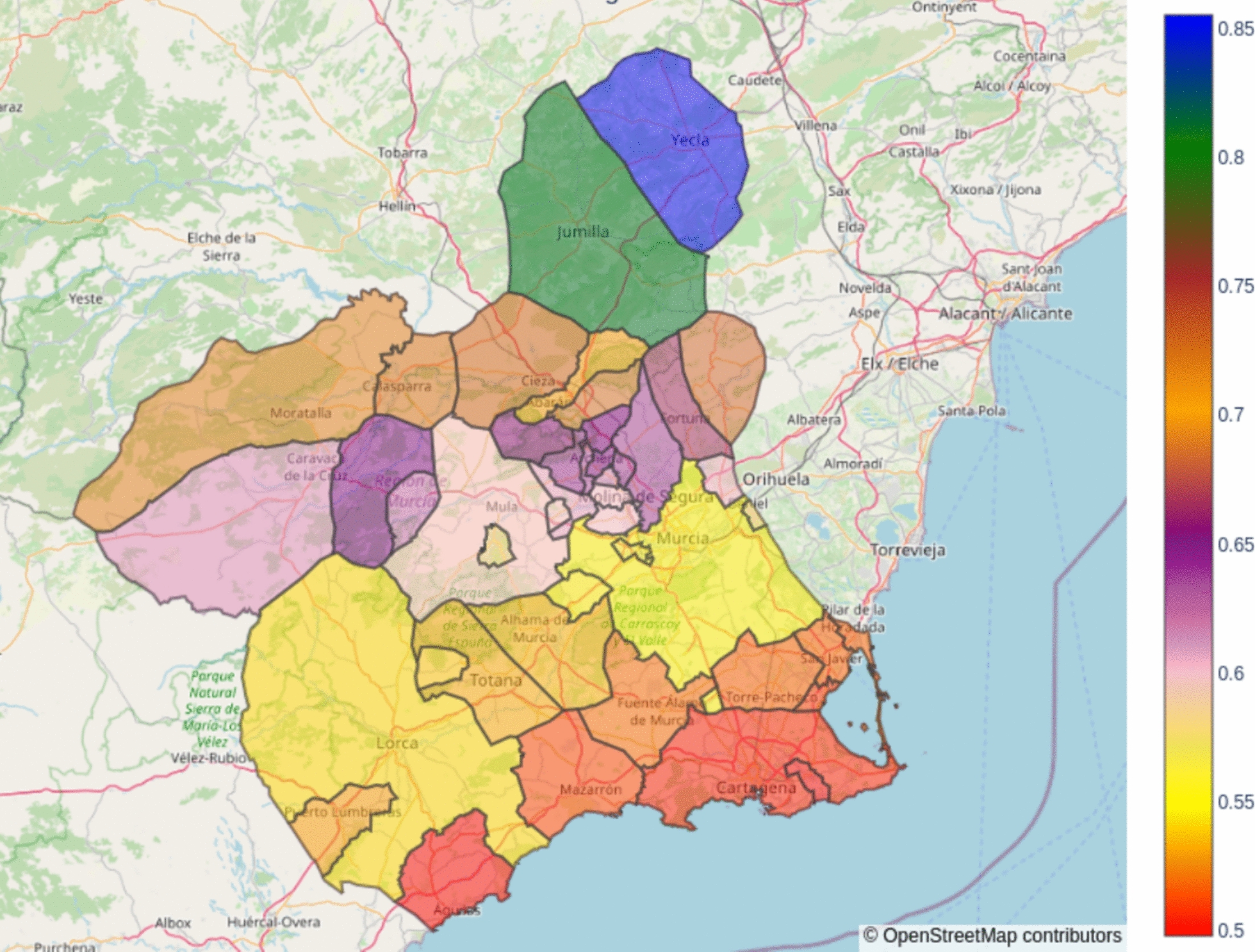
Result of the heatmap query, showing the spatial distribution of CH_4_ over different municipalities in μg/m^3^

The *heatmap* query can provide concentration values for each one of the municipalities for a concrete timestamp and pollutant. Thus, it is possible to represent spatial heatmaps to understand the dispersion of pollutants, one of the main insights of chemistry transport models. An example is shown in Fig. 12, where the CH_4_ heatmap corresponding to the timestamp 2023-12-26 06:00:00 in UTC. It is observed to have homogeneity in the domain, with the maximum values in the north of the region, where the agriculture and livestock activities related to methane emissions are more frequent.

–Counting of the number of superations in a time range

**Fig. 13:**
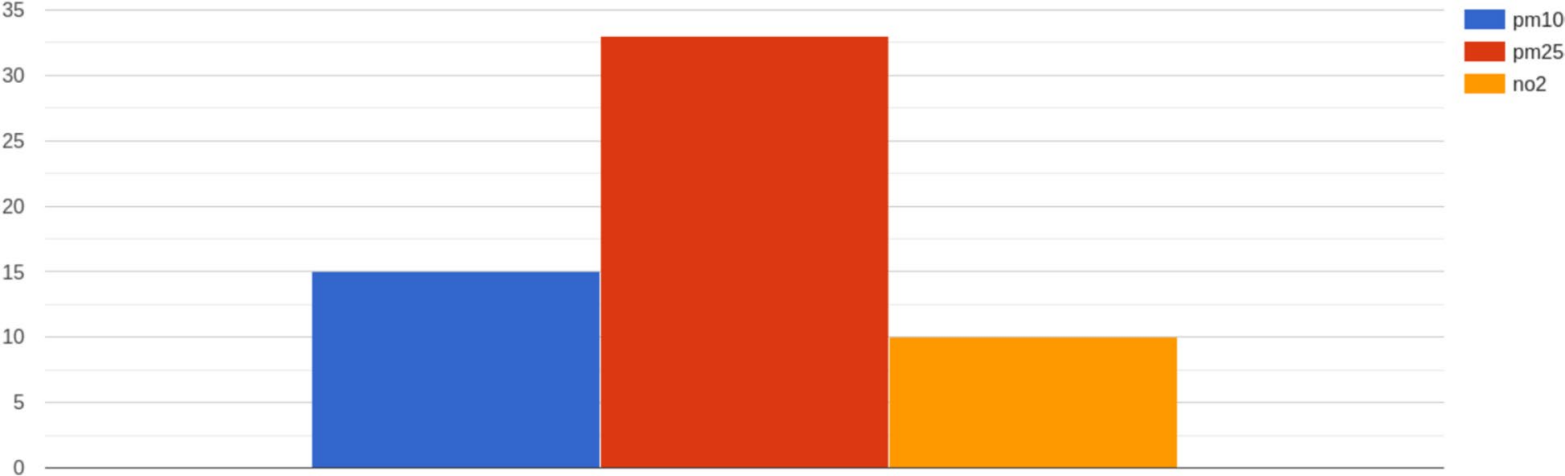
Result of the superations query, showing the frequency in number of occurrences of a superation of PM10, PM2.5 and NO_2_

After applying the *superations* query, it is possible to plot an histogram with the number of superations per pollutant (Fig. 13). In this example, the histogram only plots PM10, PM2.5 and NO_2_ because they are the pollutants presenting superations of the umbral values defined in *Materials and Methods*. In concrete, this example is executed for the municipality of Cartagena from 2023-12-26 06:00:00 to 2023-12-28 06:00:00, in UTC. The results shown 15 superations for PM10, 33 superations for PM2.5 and 10 superations for NO_2_.

–Calculation of the Air Quality Index (AQI) for a concrete timestamp

**Fig. 14:**
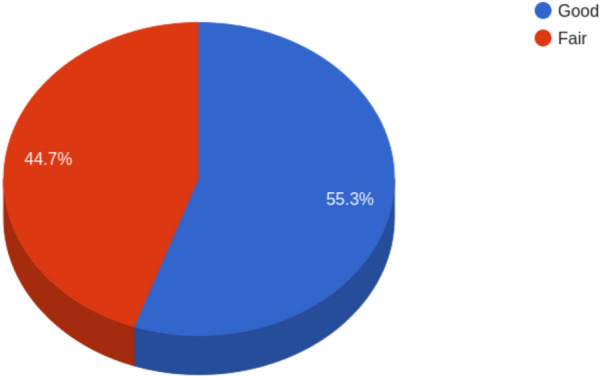
Result of the air quality index query, showing the percentage of each one of the status during 72 h. In this example, only good and fair forecasting are found

Last, the *air quality index* query could retrieve successfully the requested information, as presented in Fig. 14. The air quality index for 72 forecasts—from 2023-12-26 06:00:00 to 2023-12-28 06:00:00—has been calculated and aggregated to compute percentages in the municipality of Murcia. It is important to note that only *Good* and *Fair* forecasts have been found because the next levels occur with extreme values that are not usual. This query demonstrate that our knowledge model allows to extract information from chemical entities and aggregates per forecast, as well as apply complex operations and queries.

## Discussion

Knowledge graph representation is a trend in big data and scientific computation, because of the ability to keep semantic information in large datasets [56]. However, this strategy has not been so much explored in the air quality field, and only there are some preliminary approaches [57]. For this reason, this paper has proposed an ontology-driven knowledge graph from air quality simulations, levering researchers to connect air quality data graphs through ontologies, and increasing FAIRness across the European air quality network as demanded by the Directive 2024/2881/EC.

First, we hypothesized that the netCDF files obtained from the CHIMERE-WRF model could be transformed into RDF triples, so that an ontology is providing the classes and properties for describing the data, and that this process could be done with the support of state-of-the-art tools. Taking into consideration the results presented, we can conclude that the hypothesis has been validated for short term air quality forecasting by using SWIT. An RDF file in *turtle* format has been obtained, and this can be automated for a simulation of 72 h, generating a knowledge graph of 56.1 MB including 226,269 triples and 69,056 individuals. In more detail, Table 5 demonstrates that the knowledge graph keeps the cardinality constraints shown in Fig. 4*.* In 73 h, for each *AirQualityForecast* individual there is a unique individual of each chemical compound—there is a total 3825 of *AirQualityForecast* individuals and 3825 for each one of the chemical species. The only exception is PM10, which presents a total of 7650 individuals because PM10 also includes the PM2.5 individuals. Our analysis reveals the following findings: (i) the ontology is conceptually well designed; (ii) the mapping has been done properly, as the knowledge graph keeps these domain-specific rules; and (iii) it is possible to infer that for any chemical compound—excluding particles, as they are not chemical compounds—, the number of individuals is equal to the number of *AirQualityForecast* individuals.

In addition, Fig. 8 demonstrates that we can generate bigger graphs. In this sense, a scalability analysis has been performed, obtaining a linear complexity with R^2^ = 1.00 when individuals increase, which suggests that our approach could be extended to long term simulations as a future research line. The p-value = 2.67e-23 indicates that the significance of these results is high, and that *Algorithm 1* will keep this linearity for larger graphs. Similar results are obtained regarding the evolution of size with number of individuals, with R^2^ = 0.999 and p-value = 8.09e-18. This is coherent with the complexity analysis performed in the state of the art, which assesses that in the best scenario (without collisions between individuals) the complexity of SWIT is linear [37]. In addition, this analysis is trustable because of the low variability and the number of tests presented in Fig. 7.

This transformation allows storing the triples in a GraphDB database providing an SPARQL endpoint. We have achieved not only the storage of the air quality simulations in an RDF database but also access to the data in a proper way. This is an alternative to the traditional relational and time series approach, which present limitations regarding the flexibility to represent complex relationships in air quality data because of the underlying complex schemas. Some studies have implemented graph databases for air quality data, but more focused on sensor measurements than air quality forecasting or model simulations [58]. RDF stores have been used in urban digital twins, one of the main applications of air quality modeling, which investigates the relationship between traffic flows and air quality conditions through a chain of complex simulation models [59].

Furthermore, the triples generated are consistent according to the results presented in Figs. 9 and 10. Thus, it is possible to extract relevant information for the stakeholders as the timeseries for one municipality, the heatmap for a concrete heatmap, counting the number of superations, subset data for further data analysis or compute the air quality index. All these operations can be done with the same precision as in relational databases, demonstrating that the use case requirements defined in *Description of the Use Case* can be satisfied. This consistency is also a proof that the knowledge graph generation process has been performed properly, and information in the netCDFs is conserved after the mapping, showing that the hypothesis is validated not only in a formal point of view, but also in terms of the content. Further research will focus on proving that our method is unbiased, which is a challenging task.

One of the reasons for the successful mapping is the use of a proper ontology. In this work, a new ontology has been defined to model a CHIMERE simulation, providing a new insight in the state of the art, and complaining with the second objective proposed for this paper. In addition, this ontology meets with the quality standard according to the OQuaRE metrics. To interpret these results, we followed the recommendations stated by OQuaRE authors, which are collected in Table 8 [45]. It is important to note that this table collects only some of the metrics computed in Table 3, which are related with the modularity, reusability, analyzability, changeability, stability and testability of the ontology. The methodology consists of assigning a + + if the metric contributes to this characteristic with a score of 5, a + if the score is 3 or 4, and a minus if the score is less than 3. The only exception is the relation of NOCOnto with reusability, which decreases for high scores of this metric.

**Table 8:** Quality metrics interpretation

Charasteristic	WMCOnto	DITOOnto	NOCOnto	RFCOnto	NOMOnto	LCOMOnto	CBOOnto
Modularity	+ +						+ +
Reusability	+ +	+	−	+ +	+ +		+ +
Analysability	+ +	+		+ +	+ +	+	+ +
Changeability	+ +	+	+	+ +	+ +	+	+ +
Modification Stability	+ +		+	+ +		+	+ +
Testability	+ +	+		+ +	+ +	+	+ +
Value	2.6	5.0	2.8	2.8	1.9	2.6	1.0

In general, results in Table 3 shows that the ontology complies with all the quality standards, and with all the characteristics analyzed. The OQuaRE results also reveal that the ontology is well-annotated, logically structured, and appropriately deep, with strong relationships and properties supporting its semantic expressiveness and interoperability. The consistently high scores across most metrics validate its quality and scalability for use in expert systems, particularly in air quality monitoring and management. In concrete, the ontology achieved high scores for both ANOnto and AROnto, with ANOnto scoring 5 (> 80%) and AROnto scoring 4 (60–80%), indicating that the classes in the ontology are well-annotated and adequately described with attributes, enhancing clarity and usability. Regarding class relationships, the CHIMERE ontology achieves the highest score of 5 for CBOOnto, CBOnto2, NACOnto, NOCOnto, TMOnto, TMOnto2, and POnto, pointing out a well-balanced hierarchy and the appropriate use of parent–child relationships. The DITOnto metric scored 3 (4–6), indicating that the ontology has a moderately deep hierarchical structure, while the LCOMOnto score of 4 (2–4) shows that the ontology maintains an appropriate level of conceptual relatedness, promoting the separation of concerns and ensuring the independence of components. Last, the high score of 5 (60–80%) for INROnto indicates that the ontology is designed to include a meaningful number of relationships per class, facilitating its ability to integrate with external datasets and ontologies.

The only negative point is regarding reusability because of the high score in NOCOnto. In any case, we interpreted that this is composed by the high reusability provided by other metrics such as WMCOnto, DITOOnto, RFCOnto, NOMOnto or CBOOnto. However, it is true that some metrics have negative scores in Table 3, and it is necessary to discuss why these scores are obtained. This is the case of CROnto and RROnto, with scores equal to 1 and 2, respectively. Regarding CROnto, we obtained a score equal to one. This is an expected result which does not have to be interpreted negatively from the perspective of the quality of the ontology, because this metric accounts for individuals, which do not exist in our ontology. Concerning RROnto, a result equals to 2 means that the ontology does not have many datatype properties and object properties, which is justified by the low complexity of pollutant relationships [50]. These results mean that the ontology can be used for other members of the CHIMERE and scientific community.

This ontology has been also compared with others present in the state of the art, as shown in Fig. 5. From these results, it could be concluded that the CHIMERE ontology is more similar to the chemical ontologies than the environmental ones. This is logical because this ontology is focused on describing chemical components in the atmosphere as well as mathematical processes in simulations, and we have reused terms from CHEBI to describe the chemical contaminants. However, the ontology has a separated branch in the dendrogram suggesting that it covers a knowledge domain not explored in the state of the art. The most similar approach found in the literature is the OntoKin ontology, which models chemical kinetic reactions mechanisms [35]. However, the CHIMERE ontology improves the OntiKin approach as this ontology only covers chemical reaction while a chemistry transport model includes other physicochemical processes as transport, deposition and emissions. For this reason, one insight that we extracted from this work is the need to continue researching in chemistry transport models ontologies. A limitation of the proposed solution is that the ontology only covers the CHIMERE domain, while there are several chemistry transport models in the state of the art [60]. Thus, we cannot affirm that the proposed ontology works for mapping data from other models even though the ontology has been designed using common vocabularies. The reason is that chemistry transport models are quite heterogeneous in processes involved, chemical compounds covered, resolution and applications as well as file format processing.

The proposed knowledge model makes a significant contribution in terms of standardization and interoperability, as using knowledge graphs and SPARQL present the following advantages: (i) the complexity of the SPARQL queries is lower in comparison to retrieve data from a netCDF file and (ii) the data retrieved using this approach is semantically annotated making them more FAIR. In this sense, the evaluation of the knowledge graph through competency questions demonstrated its ability to address key domain-specific tasks relevant to air quality monitoring and management, assuring that the mapping has been done correctly from the netCDF to knowledge graph.

Finally, it is important to highlight the limitations of the proposed solution. The main one is the application only to short term forecasting, understood as simulation of no more than 73 h. It is true that this kind of simulations cover the requirements of the use case proposed, but there are some applications that require long simulations of a whole year, such as air quality zoning [61] or spatial feature extraction for convolutional networks training [62]. In addition, Directive 2008/50/EC defines a maximum of annual superations for certain concentration thresholds, that can only be provided to stakeholders. However, address long term simulation is challenging from the computational point of view. First, because running long-term simulations, especially at high resolutions, is challenging and requires long computations. Secondly, the mapping itself with SWIT can be also complex even though the computational complexity is linear. The reason is the preprocessing of extracting data from netCDF to generate the csv, and then the XML. The problem is that netCDF size increases exponentially to GB order or more, being difficult to extract data from the files as the disk I/O times increases. In such a case, the HPC techniques implemented in SWIT may contribute to speed up the process.

## Conclusions

In this work, we have developed and evaluated an automatic knowledge graph generation system for CHIMERE simulations, demonstrating that netCDF files can be transformed into RDF triples by using SWIT software, an ontology and a set of mapping rules, being able to populate knowledge graphs with new individuals with a linear computational complexity, making it scalable for short term simulations. In addition, the knowledge graphs generated were stored in a GraphDB database, from where stakeholders can extract relevant information through the SPARQL endpoint. Moreover, we have provided a new ontology describing the CHIMERE model, highlighting the need for new modeling semantic approaches in this field, which is not covered in the ontologies present in the state of the art. Additionally, we have shown that our ontology meets the quality requirements being reusable, modulable, adaptable, testable, modifiable and updatable.

However, the results obtained open new research possibilities and highlight different limitations that should be addressed in different studies: (i) the limitation of the ontology to the CHIMERE model, and the importance to extend it to other chemistry transport models; (ii) apply our methodology to long term simulations, covering time periods of years and (iii) improve the efficiency of netCDF file processing, especially regarding the disk I/O times. On the other hand, more research is needed in the field of air quality knowledge to understand the role of neural networks in making better decisions and predictions. Last, this methodology should include interaction and expert feedback to include human knowledge in the interpretation and requirements design.

## Data Availability

Source codes and example datasets are available on the GitHub repository https://github.com/eduardoillueca/chimere-ontology. This includes the owl files with the ontology as well as the SPARQL queries and required scripts for processing. The CHIMERE source code can be accessed from https://www.lmd.polytechnique.fr/chimere/Source codes and example datasets are available on the GitHub repository https://github.com/eduardoillueca/chimere-ontology. This includes the owl files with the ontology as well as the SPARQL queries and required scripts for processing. The CHIMERE source code can be accessed from https://www.lmd.polytechnique.fr/chimere/.
